# Trimetazidine Use and the Risk of Parkinsonism: A Nationwide Population-Based Study

**DOI:** 10.3390/ijerph17197256

**Published:** 2020-10-04

**Authors:** Seungyeon Kim, Yun Mi Yu, Jeongyoon Kwon, Kyeong Hye Jeong, Jeong Sang Lee, Euni Lee

**Affiliations:** 1 College of Pharmacy & Research Institute of Pharmaceutical Sciences, Seoul National University, Seoul 08826, Korea; gsy92@snu.ac.kr (S.K.); pp77girl@snu.ac.kr (J.K.); 2 Department of Pharmacy and Yonsei Institute of Pharmaceutical Sciences, College of Pharmacy, Yonsei University, Incheon 21983, Korea; yunmiyu@yonsei.ac.kr; 3 Department of Pharmaceutical Medicine and Regulatory Sciences, Colleges of Medicine and Pharmacy, Yonsei University, Incheon 21983, Korea; 4 College of Pharmacy, Chung-Ang University, Seoul 06974, Korea; jnkh7@cau.ac.kr; 5 Department of Thoracic & Cardiovascular Surgery, SNU-SMG Boramae Hospital, Seoul 07061, Korea; 6 Department of Thoracic & Cardiovascular Surgery, College of Medicine, Seoul National University, Seoul 07061, Korea

**Keywords:** trimetazidine, parkinsonism, drug-induced parkinsonism

## Abstract

An association between trimetazidine (TMZ), an anti-anginal drug, and parkinsonism has been reported in a number of studies. However, evidence from studies with long-term follow-up and better validity is lacking. We investigated the risk of TMZ-associated parkinsonism, specifically the incidence rate, cumulative dose–response relationship, and combined effects with other parkinsonism-inducing medications. This propensity score-matched retrospective cohort study was conducted using 14-year health insurance claims data in South Korea. The risk of parkinsonism was evaluated using multivariate Cox proportional hazard regression analysis, adjusted for comorbidities and concurrent medications. A total of 9712 TMZ users and 29,116 matched non-TMZ users were included. TMZ users had a significantly higher incidence rate of parkinsonism than non-TMZ users (9.34 vs. 6.71 per 1000 person-years; *p* < 0.0001). TMZ use significantly increased the risk of parkinsonism (adjusted hazard ratio = 1.38; 95% confidence interval = 1.26–1.51). Increased risks were observed with accumulated doses of TMZ, as well as concurrent use of other parkinsonism-inducing medications. The findings indicate that TMZ use significantly increases the risk of parkinsonism in the South Korean population. Closer monitoring should be considered for TMZ users, especially for those who are older, using TMZ at high cumulative doses and other parkinsonism-inducing medications.

## 1. Introduction

Parkinson’s disease (PD) is the second most common neurodegenerative disorder, with a growing prevalence worldwide and about 7% of cases were reported to develop parkinsonism as a result of drug-induced adverse effects [1]. Drug-induced parkinsonism (DIP)—a secondary parkinsonism—is defined as the development of parkinsonian symptoms after being treated with dopamine-blocking or -depleting drugs and accounts for 9–20% of patients with parkinsonism [2,3]. Unlike idiopathic Parkinson’s disease (iPD), DIP often shows different clinical characteristics, including symmetrical features and female predominance [4], but is not easily distinguishable. Numerous medications, such as typical and atypical antipsychotics, prokinetics, and some calcium channel blockers (CCBs) and anti-epileptics, have been shown to increase the risk of developing DIP [5,6]. However, trimetazidine [1-(2,3,4-trimethoxybenzyl)-piperazine dihydrochloride] (TMZ)-associated parkinsonism has not been mentioned in the literature [5,6,7].

TMZ is a metabolic regulator that promotes glucose oxidation by blocking the mitochondrial long-chain 3-ketoacyl coenzyme. It is widely used in Europe and Asia to treat angina pectoris, dizziness, and tinnitus, with documented efficacy and safety [8]. However, reversible parkinsonism was initially reported in eight TMZ users [9], and since then, a larger observational study also identified TMZ-induced parkinsonism, gait disorders, and tremor [10]. A recent study [7] documented that TMZ-associated parkinsonism showed mild clinical features and symmetrical characteristics, consistent with those of DIP [4].

In light of accumulating reports of the adverse neurological effects of TMZ, the European Medicines Agency re-evaluated the safety of TMZ, resulting in the recommendation to restrict the use of TMZ to add-on therapy only, for the symptomatic treatment of stable angina pectoris [11]. Although other parkinsonism-inducing medications have been investigated in a number of large-scale epidemiological studies, only a few have examined TMZ-associated parkinsonism, and even fewer have used population-based data and robust study designs. 

In this study, we conducted a retrospective cohort evaluation using 14-year follow-up data from South Korea’s national claims database to investigate the association between the use of TMZ and the risk of developing parkinsonism. We specifically analyzed the incidence rate, cumulative dose–response relationship, and combined effects of TMZ with concurrent use of other parkinsonism-inducing medications.

## 2. Materials and Methods 

### 2.1. Data Sources and Study Design

A retrospective cohort study was conducted using data from the National Health Insurance Service—National Sample Cohort (NHIS-NSC) 2.0, between 2002 and 2015. The NHIS is a single-payer healthcare system, providing universal health insurance coverage to all South Korean citizens, and generates research-ready cohort databases from insurance claims with selected demographic (age, sex, and insurance type) and clinical information. The NHIS-NSC database comprised about 2.2% of the total eligible Korean population in 2006, who were selected by a systematic stratified random sampling method, considering age, sex, eligibility status, and income level, as a representative sample of the general South Korean population. The database was constructed by following selected participants retrospectively for 4 years (2002–2005) and prospectively for 10 years (2006–2015) [12]. 

The individual records from the NHIS-NSC data were anonymized and de-identified by the Health Insurance Review and Assessment Service, mandated by the Personal Information Protection Act and National Health Insurance Act, prior to researchers accessing the data. All research was performed in accordance with the ethical requirements of the Seoul National University Institutional Review Board (IRB) and approved by the IRB without the requirements for obtaining written informed consent (IRB No. E1808/003-011). 

Adult individuals aged over 19 years in 2002 (*n* = 725,224) were selected from the NHIS-NSC database. The study period, which included the collection of longitudinal data from January 2002 to December 2015, was divided into two periods on either side of an index date (1 January 2008) as: (1) “exposure ascertainment period” (January 2002 to December 2007); and (2) “follow-up period” (January 2008 to December 2015). We collected data on sociodemographic characteristics, prescriptions of TMZ and any concurrent medications, and comorbid clinical conditions during the “exposure ascertainment period”. The study outcome—a new event of parkinsonism—was captured during the “follow-up period”. 

### 2.2. Data Collection

New TMZ users were identified during the identification period, which was from January 2006 to December 2007, and participants who were prescribed TMZ for the first time before or after this period (*n* = 51,823) were excluded. To evaluate the cumulative dose–response relationship, the accumulated dose of TMZ was calculated by multiplying the TMZ dosage, frequency, and days’ supply. We used the defined daily dose (DDD), which was developed by the World Health Organization (WHO), to convert accumulated doses into cumulative DDD (cDDD) [13]. Depending on the level of cumulative doses, TMZ users were divided into three groups: (1) ≤7 cDDDs; (2) 7–30 cDDDs; and (3) >30 cDDDs.

**Figure 1:**
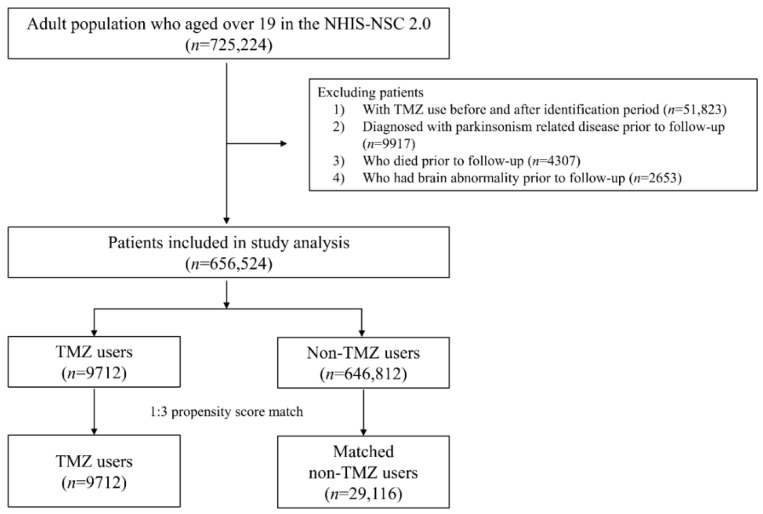
Flow chart of study cohort. TMZ—trimetazidine.

Participants with diagnoses of extrapyramidal or movement disorders (*n* = 9917) or who had died (*n* = 4307) prior to the follow-up period were excluded based on the following International Classification of Diseases, Tenth Revision (ICD-10): G20-G26. Furthermore, participants with a government-rated disability grade due to a brain abnormality prior to the follow-up period (*n* = 2653) were also excluded, since brain abnormality is a documented risk factor for parkinsonism [14]. A flow chart depicting the selection procedure is shown in Figure 1.

The study outcome was a new occurrence of parkinsonism during the follow-up period, which was identified and confirmed when a patient was recorded with the following ICD-10 diagnostic codes at least three times, in either an inpatient or outpatient setting: G20, G21.1, G21.2, G21.8, G21.9, and G24-G26. 

Sociodemographic characteristics, including the patient’s age, sex, area of residence, insurance type (i.e., Health Insurance or Medical Aid), and death record, were collected. The presence of comorbid diseases and concurrent medications known to increase the risk of parkinsonism from the literature review were also identified [15,16,17]. Comorbid conditions included diabetes mellitus, end-stage renal disease (ESRD), stroke, dementia, hypertension, ischemic heart disease, dyslipidemia, head injury, and severe liver disease [15,16,17]. Concurrent medications included typical and atypical antipsychotics, prokinetics, CCBs, anti-epileptics, and dopamine depleters [5,6]. ICD-10 codes for the comorbid diseases and details of concurrent medications are shown in Supplementary Materials Tables S1 and S2. 

### 2.3. Statistical Analysis

The propensity score (PS) for TMZ use was estimated using a multivariate logistic regression model with consideration of predictor variables, including sex, age, insurance type, area of residence, comorbid diseases, and concurrent use of parkinsonism-inducing medications. The predictability of the constructed PS model was high, with a *c*-statistic of 0.81 (Figure S1). Non-TMZ users were matched to TMZ users in a ratio of 3:1 by greedy matching, with a caliper of 0.2 times the standard deviation (SD) of the logit PS. This matching process resulted in similar PS distributions between TMZ users and non-TMZ users (Figure S2).

We used descriptive statistics to summarize the characteristics of the study population. Pearson’s Chi-squared test and the Student’s t-test were used for categorical and continuous variables, respectively, to compare baseline characteristics between TMZ users and matched non-TMZ users. The incidence rate per 1000 person-years was calculated by dividing the number of parkinsonism events by the total number of person-years at risk, and multiplying by 1000. Accumulated person-years at risk were computed from the index date to either date of diagnosis of parkinsonism, death, or 31 December 2015, whichever occurred first. The risk of parkinsonism was assessed using univariate and multivariate Cox proportional hazard regression analyses to adjust the effects of demographic characteristics, comorbidities, and concurrent medications, and hazard ratios (HRs) and 95% confidence intervals (CIs) were estimated. To confirm the proportional hazard assumption, log (minus log) curves were examined (Figure S3). The cumulative dose–response relationship between TMZ and the risk of parkinsonism, as well as the combined effects of TMZ with concurrent use of other parkinsonism-inducing drugs, were also evaluated by Cox proportional hazard modeling.

Sensitivity analysis was conducted by shifting the index date from 1 January 2008 to 1 January, 2007, 2009, or 2010. As the index date shifted, the “exposure ascertainment period” and “follow-up period” were changed accordingly, and the eligible populations, as well as variables related to exposure, outcome, comorbid diseases, and concurrent medications were also reassessed. Accordingly, any changes in the risk of parkinsonism were evaluated by calculating the HRs. All statistical analyses were performed using SAS version 9.4 (SAS Institute Inc., Cary, NC, USA), and the level of statistical significance was set at *p* < 0.05.

## 3. Results

Of the eligible 656,524 patients in the study cohort, 9712 TMZ users and 29,116 matched non-TMZ users were analyzed in this study (Figure 1). Baseline demographic characteristics of the study population of TMZ users and matched non-TMZ users are shown in Table 1. The mean age (±SD) of the study population was 52.22 (±15.15) years, and women constituted 65.29%. About 44% of the study population lived in urban areas. No statistical differences were observed between TMZ and non-TMZ users with respect to age, sex, or area of residence. 

A total of 2084 parkinsonism events occurred during the observation period of 282,654 person-years, with an overall incidence rate of 7.37 per 1000 person-years. The incidence rate of parkinsonism was significantly higher in TMZ users than matched non-TMZ users (9.34 vs. 6.71 per 1000 person-years, respectively; *p* < 0.0001), and TMZ use significantly increased the risk of parkinsonism (adjusted HR [aHR] = 1.38; 95% CI = 1.26–1.51). Being male and having Medical Aid insurance were associated with a reduced and an increased risk of parkinsonism, respectively. Compared to the population who were aged under 50 years, those aged over 50 years were more likely to develop parkinsonism (aHR = 2.48; 95% CI = 2.18–2.81 for age 50–64 years, and aHR = 3.22; 95% CI = 2.81–3.68 for age 65 years and older). Comorbidities including diabetes, ESRD, and stroke, and concurrent medications such as typical antipsychotics, atypical antipsychotics, prokinetics, and CCBs were significant predictors of a new diagnosis of parkinsonism. Full model estimates are shown in Table 2. No covariate showed any significant interaction with TMZ use by multivariate Cox proportional hazard regression modeling (Supplementary Materials Table S3). 

**Figure 2:**
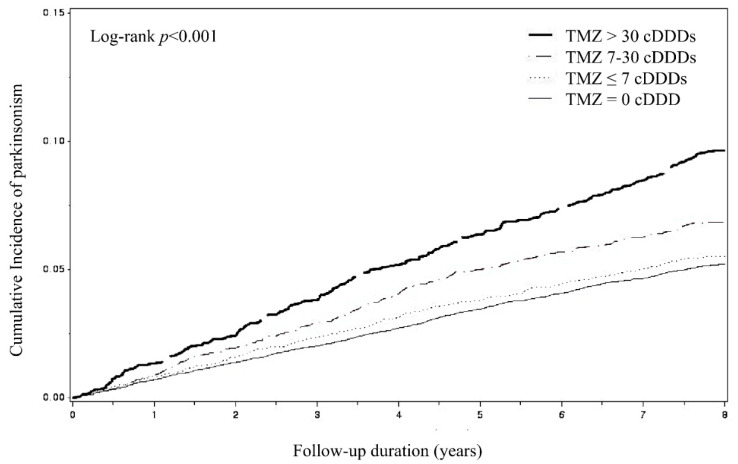
Kaplan–Meier curve with cumulative incidence of parkinsonism by cumulative defined daily doses (cDDDs) of trimetazidine use during follow-up period. TMZ—trimetazidine.

Figure 2 shows the Kaplan–Meier curve for cumulative incidence of parkinsonism. Cumulative incidence increased with higher cumulative doses of TMZ, compared with non-TMZ use (*p <* 0.001). The combined effects of TMZ and concurrent medications, such as antipsychotics, prokinetics, CCBs, anti-epileptics, and dopamine depleters, on the risk of parkinsonism are shown in Table 3. TMZ users who were concurrently on other medications had a higher risk of parkinsonism than TMZ users who were not on other medications. The risk of parkinsonism increased as the number of concurrent medications increased (aHRs = 2.30, 2.96, and 4.58; 95% CIs = 1.80–2.93, 2.27–3.86, and 3.16–6.64 for one, two, and three concurrent medications, respectively).

Sensitivity analysis showed that the risk of parkinsonism associated with TMZ use for each shifted index date remained higher compared with non-TMZ use (Supplementary Materials Table S4). 

## 4. Discussion

To the best of our knowledge, this is the first population-based cohort study with longitudinal follow-up demonstrating the association between TMZ use and increased risk of parkinsonism in the Korean population. We recently reported a similar association between TMZ use and parkinsonism in a cross-sectional study using 1-year data [18]. Compared with our previous cross-sectional study, the present study has greater validity because we used a cohort study design, included a greater number of TMZ users (>9000 patients), and focused on the long-term effects of TMZ (8-year follow-up period). Moreover, the cumulative dose–response relationship between TMZ use and the risk of parkinsonism, as well as the combined effects of TMZ with other parkinsonism-inducing medications were also demonstrated using individual prescription records. Since TMZ-associated parkinsonism is highly unrecognizable from healthcare professionals and usually neglected in the literature [5,6,7], the findings from our study using real-world data derived from the general population could provide clinical relevance and insights on the occurrence of TMZ-associated parkinsonism, adding to existing knowledge regarding the risk of TMZ itself or in combination with other drugs.

Our major findings indicated a significantly higher incidence rate and an increased risk of parkinsonism in TMZ users than in non-TMZ users. The level of the association with TMZ in our study (aHR = 1.38) appears to be similar to the adjusted odds ratio (aOR) from a cross-sectional evaluation using 1-year data (aOR = 1.39) [18]. This suggests that the length of follow-up may not have significantly influenced the risk of parkinsonism. Consistent with many previous studies, our regression results showed that antipsychotics and prokinetics were associated with a higher risk of parkinsonism than that with anti-epileptics and CCBs, which are considered intermediate risk drugs [5,19]. The risk of parkinsonism may be lower with TMZ compared to high risk drugs, such as antipsychotics and prokinetics, but higher with TMZ than intermediate risk drugs. 

Of the various potential pathways of developing DIP [20], a possible mechanism underlying TMZ-induced parkinsonism may be blockade of post-synaptic dopamine D_2_ receptors, which are mainly located in the striatum [7]. Both in vitro and in vivo studies have demonstrated blockade of D_2_ receptors by certain drugs with the piperazine structure, such as chlorpromazine, cinnarizine, and TMZ [21,22]. Since the affinity for D_2_ receptors is dependent on the chemical structure of piperazine ligands [22,23] and may contribute to developing parkinsonism [24], further investigation into the affinities of receptors for TMZ and its metabolites is necessary.

In the present study, we determined the cumulative dose–response relationship by analyzing all reliable prescription claims data generated from both outpatient and inpatient encounters. The incidence rate of parkinsonism showed an increasing trend with cumulative dose of TMZ, from even the lower cumulative doses (≤7 cDDDs) of TMZ compared with no use of TMZ. This dose–response relationship has been similarly reported in previous studies of other drugs, such as flunarizine and zolpidem [25,26]. An in vitro study suggested that the accumulation of metabolites [22] could be a potential explanation for the cumulative dose–response relationship of flunarizine. We believe that healthcare professionals should be aware of the cumulative dose effect of TMZ on the risk of parkinsonism and should exercise caution when prescribing TMZ for an extended period of time.

Our study demonstrated the combined effect of TMZ with other parkinsonism-inducing drugs. Patients who used TMZ combined with three or more parkinsonism-inducing drugs showed almost twice the risk of parkinsonism than those who used TMZ plus one of these drugs. This finding has particular significance for older patients subjected to polypharmacy, as they are more frequently prescribed parkinsonism-inducing medications, including antipsychotics, prokinetics, and CCBs, to treat multiple chronic conditions and decreased physiological functioning [27,28]. A study in older Korean patients found that, on average, more than three antipsychotic drugs were prescribed concurrently in psychiatric outpatient settings [29]. Since age is also considered an obvious risk factor for parkinsonism [6], closer monitoring of older patients for DIP is essential.

The literature indicated that TMZ-associated parkinsonism impacted patients’ quality of life and was poorly responsive to clinical treatment with levodopa or dopamine agonists due to the pathogenic mechanisms of DIP [7]. As DIP is a mostly reversible condition, withdrawal of the offending drugs improved the condition [4,5,7], and some drugs including TMZ could unmask or worsen the premature iPD [30]. Although early recognition and prompt discontinuation of such drugs can be considered crucial for the management of parkinsonism, we believe the fact that TMZ has not been well documented as a parkinsonism-inducing medication in previous DIP-related studies [2,5,19] should be highlighted. Even after the indication for TMZ was restricted due to safety issues, many physicians still prescribed TMZ for vertigo and tinnitus off-label, and they were not aware of the latest safety information regarding TMZ [31]. Therefore, we believe that our study, demonstrating the significant risk of TMZ-associated parkinsonism, will help to raise awareness of TMZ-associated parkinsonism and the importance of recognizing DIP.

There are some limitations to this study. First, the diagnosis of parkinsonism in our study population was identified via ICD-10 codes taken from the claims database. This could be inaccurate or incomplete, because accurate diagnosis of TMZ-induced parkinsonism should be based on imaging investigation, together with demonstrable improvement of clinical features upon TMZ discontinuation [20]. In addition, data on the potential confounders of parkinsonism, including genetic factors, toxic substances, certain infectious diseases, and lifestyle factors, could not be obtained from the claims database [2,6]. Second, there could have been selection bias when identifying non-TMZ users from the NHIS-NSC 2.0. To minimize this bias, we used a PS-matching process, and baseline characteristics, except for some comorbid conditions and concurrent medications, showed no significant differences between TMZ users and non-TMZ users. Third, treatment duration or cumulative doses of other concurrent medications were not considered in our analysis. Since both the duration and the dose of the offending medication are possible risk factors for developing DIP [32], further analyses are needed. Fourth, there could be possible “TMZ exposure misclassification” of patients, especially in cases where patients started TMZ after the “exposure ascertainment period”. However, to address this issue, we performed sensitivity analysis by shifting the index date backward and forward, which resulted in only minor changes in the aHRs. We believe additional comparative evaluations are needed in the future to better understand the risk of parkinsonism with other populations as the patterns of drug use, regulatory requirements, or treatment guidelines might differ by countries. 

## 5. Conclusions

Our 14-year longitudinal cohort study showed that TMZ use was associated with an increased risk of parkinsonism in the Korean population. Our study highlights the need for increased awareness of TMZ-associated parkinsonism and the importance of close monitoring, especially in older patients who may require high cumulative doses of TMZ concurrently with other parkinsonism-inducing medications.

## Figures and Tables

**Table 1:** Demographic characteristics of the study population.

Characteristics	Total		Trimetazidine Users		Matched Non-Trimetazidine Users		*p*-Value *
	n = 38,828	%	n = 9712	%	n = 29,116	%	
Sex							0.31
Male	13,477	34.71	3412	35.13	10,065	34.57	
Female	25,351	65.29	6300	64.87	19,051	65.43	
Age							
Mean (SD)	52.22 (15.15)		51.98 (15.21)		52.30 (15.13)		0.08
Younger than 50 years	16,460	42.39	4190	43.14	12,270	42.14	0.17
50–64 years	12,797	32.96	3182	32.76	9615	33.02	
65 years and older	9571	24.65	2340	24.09	7231	24.84	
Insurance type							0.002
Health Insurance	35,622	91.74	8835	90.97	26,787	92.00	
Medical Aid	3206	8.26	877	9.03	2329	8.00	
Area of residence							0.77
Capital city (Seoul)	6088	15.68	1509	15.54	4579	15.73	
Metropolitan city	10,955	28.21	2766	28.48	8189	28.13	
Rural area	21,785	56.11	5437	55.98	16,348	56.15	
Comorbidity							
Diabetes	14,334	36.92	3554	36.59	10,780	37.02	0.45
End stage renal disease	140	0.36	41	0.42	99	0.34	0.25
Stroke	10,218	26.32	2617	26.95	7601	26.11	0.10
Dementia	89	0.23	27	0.28	62	0.21	0.26
Hypertension	21,423	55.17	5241	53.96	16,182	55.58	0.006
Ischemic heart disease	11,817	30.43	2981	30.69	8836	30.35	0.52
Dyslipidemia	18,052	46.49	4478	46.11	13,574	46.62	0.38
Head injury	1208	3.11	342	3.52	866	2.97	0.008
Severe liver disease	572	1.47	164	1.69	408	1.40	0.04
Concurrent Medication ^†^							
Typical antipsychotics	2746	7.07	738	7.60	2008	6.90	0.02
Atypical antipsychotics	506	1.30	148	1.52	358	1.23	0.03
Prokinetics	32,901	84.74	8172	84.14	24,729	84.93	0.06
Calcium channel blockers	9178	23.64	2369	24.39	6809	23.39	0.04
Anti-epileptics	551	1.42	167	1.72	384	1.32	0.005
Dopamine depleters	5	0.01	2	0.02	3	0.01	0.60
Propensity score			0.95 (0.060)		0.95 (0.059)		0.70

* Pearson’s Chi-squared and Student’s t-test were used for comparing the categorical and continuous variables between trimetazidine users and matched non-trimetazidine users, respectively. The continuous variables in this table were the mean age and propensity score. ^†^ The complete list of concurrent medications is shown in Supplementary Materials Table S2. Abbreviation: SD—standard deviation.

**Table 2:** Incidence rates and hazard ratios of parkinsonism associated with trimetazidine use.

Characteristics	No. of Subjects	Person-Years	No. of Events	Incidence Rate (per 1000 Person-Years)	Unadjusted HRs (95% CI)	*p*-Value	^†^ Adjusted HRs (95% CI)	*p*-Value
All subjects	38,828	282,654	2084	7.37				
Trimetazidine								
No	29,116	211,814	1422	6.71	1.00 (reference)		1.00 (reference)	
Yes	9712	70,840	662	9.34	1.39 (1.27–1.53)	<0.0001	1.38 (1.26–1.51)	<0.0001
Sex								
Male	13,477	96,966	565	5.83	0.71 (0.65–0.78)	<0.0001	0.80 (0.73–0.88)	<0.0001
Female	25,351	185,689	1519	8.18	1.00 (reference)		1.00 (reference)	
Age								
Younger than 50 years	16,460	128,910	394	3.06	1.00 (reference)		1.00 (reference)	
50–64 years	12,797	94,551	882	9.33	3.05 (2.71–3.44)	<0.0001	2.48 (2.18–2.81)	<0.0001
65 years and older	9571	59,293	808	13.63	4.43 (3.93–5.00)	<0.0001	3.22 (2.81–3.68)	<0.0001
Insurance type								
Health Insurance	35,622	262,283	1759	6.71	1.00 (reference)		1.00 (reference)	
Medical Aid	3206	20,372	325	15.95	2.37 (2.10–2.67)	<0.0001	1.79 (1.58–2.02)	<0.0001
Area of residence								
Capital city (Seoul)	6088	45,254	238	5.26	1.00 (reference)		1.00 (reference)	
Metropolitan city	10,955	80,524	548	6.81	1.29 (1.11–1.51)	0.001	1.33 (1.15–1.55)	0.0002
Rural area	21,785	156,877	1298	8.27	1.57 (1.37–1.80)	<0.0001	1.42 (1.24–1.63)	<0.0001
Comorbidity								
Diabetes	14,334	99,587	1043	10.47	1.84 (1.69–2.00)	<0.0001	1.20 (1.09–1.32)	0.0002
End stage renal disease	140	729	16	21.95	2.96 (1.81–4.84)	<0.0001	2.11 (1.28–3.46)	0.003
Stroke	10,218	69,204	806	11.65	1.94 (1.78–2.12)	<0.0001	1.20 (1.09–1.31)	0.0002
Dementia	89	495	7	14.15	1.90 (0.90–3.98)	0.26	0.67 (0.31–1.41)	0.29
Hypertension	21,423	149,341	1462	9.79	2.09 (1.90–2.30)	<0.0001	1.04 (0.93–1.16)	0.55
Ischemic heart disease	11,817	81,946	850	10.37	1.68 (1.54–1.84)	<0.0001	1.09 (0.99–1.21)	0.07
Dyslipidemia	18,052	129,166	1206	9.34	1.63 (1.50–1.78)	<0.0001	1.09 (0.99–1.21)	0.08
Head injury	1208	8453	88	10.41	1.43 (1.15–1.77)	0.001	1.23 (0.99–1.53)	0.06
Severe liver disease	572	3682	39	10.59	1.44 (1.05–1.98)	0.02	1.14 (0.83–1.57)	0.42
Concurrent medication *								
Typical antipsychotics	2746	18,574	266	14.32	2.08 (1.83–2.36)	<0.0001	1.66 (1.45–1.89)	<0.0001
Atypical antipsychotics	506	2856	55	19.26	2.63 (2.01–3.44)	<0.0001	1.60 (1.21–2.10)	0.001
Prokinetics	32,901	238,121	1916	8.05	2.13 (1.82–2.49)	<0.0001	1.56 (1.33–1.83)	<0.0001
Calcium channel blockers	9178	65,308	663	10.15	1.55 (1.42–1.70)	<0.0001	1.22 (1.11–1.35)	<0.0001
Anti-epileptics	551	3526	40	11.34	1.54 (1.13–2.11)	0.01	1.00 (0.73–1.38)	0.98
Dopamine depleters	5	35	1	28.93	3.93 (0.55–27.88)	0.17	1.83 (0.26–13.08)	0.55

* The complete list of concurrent medications is shown in Supplementary Materials Table S2. ^†^ Adjusted hazard ratios (95% confidence intervals) were calculated with a multivariate Cox proportional hazard model for parkinsonism with all covariates presented in this table. Abbreviation: CI—confidence interval; HR—hazard ratio; No—number.

**Table 3:** Combined effects for parkinsonism associated with trimetazidine use and inducing medications * (*n* = 13,413).

Concurrent Medications	No. of Subjects	Person-Years	No. of Events	Incidence Rate (Per 1000 Person-Years)	Unadjusted Hrs (95% CI)	^‡^ Adjusted Hrs (95% CI)
None ^†^	3701	27,950	80	2.862	1.00 (reference)	1.00 (reference)
Trimetazidine only	1224	9206	45	4.888	1.71 (1.19–2.46)	1.70 (1.18–2.45)
Trimetazidine + 1 parkinsonism-inducing drug	5760	42,381	356	8.400	2.93 (2.30–3.73)	2.30 (1.80–2.93)
Trimetazidine + 2 parkinsonism-inducing drugs	2382	16,984	213	12.541	4.37 (3.38–5.65)	2.96 (2.27–3.86)
Trimetazidine + 3 or more parkinsonism-inducing drugs	346	2269	48	21.152	7.32 (5.12–10.48)	4.58 (3.16–6.64)

* Inducing medications included typical and atypical antipsychotics, calcium channel blockers, prokinetics, anti-epileptics, and dopamine depleters. ^†^ The study subjects who used one or more parkinsonism-inducing drugs without trimetazidine were excluded from this analysis. ^‡^ Adjusted hazard ratios (95% confidence intervals) were calculated with a multivariate Cox proportional hazard model for parkinsonism with all covariates presented in Table 2. Abbreviation: CI—confidence interval; HR—hazard ratio; No—number.

## Supplementary Materials

The following are available online at https://www.mdpi.com/1660-4601/17/19/7256/s1, Table S1: List of ICD-10 codes for comorbid disease, Table S2: List of concurrent medications, Table S3: *p*-values for the interaction terms in the multivariate Cox proportional hazard regression model between trimetazidine use and covariates, Table S4: Sensitivity analysis of adjusted hazard ratios of trimetazidine use for parkinsonism with shifting index date, Figure S1: Receiver operating characteristics (ROC) curve for the propensity score model, Figure S2: Distribution of propensity score between trimetazidine users and non-trimetazidine users before (2a) and after (2b) propensity score matching, Figure S3: Log (minus log) curves for checking proportional hazard assumption.
