# Supplementary material for: Trimetazidine Use and the Risk of Parkinsonism: A Nationwide Population-Based Study
Source: Int J Environ Res Public Health. 2020 Oct 4;17(19):7256. doi: 10.3390/ijerph17197256 (PMC7579582; doi:10.3390/ijerph17197256)
Supplement: Supplementary file 1 [file ijerph-17-07256-s001.pdf]

International Journal of
Environmental Research
and Public Health

MDPI

Supplementary Materials: Trimetazidine Use and the Risk of Parkinsonism: A Nationwide Population-Based Study

Table 1. List of ICD-10 codes for comorbid disease.

Disease	ICD-10 codes
Diabetes mellitus	E10-E14
End stage renal disease	N18.5
Stroke	I60-I69, G45, G46
Dementia	G30
Hypertension	I10-I15
Ischemic heart disease	I20-I25
Dyslipidemia	E78
Head injury	S01.9, S06.0-S06.6, S06.8, S06.9, S09.1, S09.8, S09.9
Severe liver disease	I85.0, I85.9, I86.4, I98.2, K70.4, K71.1, K72.1, K72.9, K76.5-K76.7

Abbreviation: ICD; International Classification of Disease 10th Revision.

Table S1. List of concurrent medications.

Drug Class	Drug name
Typical Antipsychotics	Chlorpromazine<br>Chlorprothixene<br>Flupentixol<br>Haloperidol<br>Levomepromazine<br>Perphenazine<br>Pimozide<br>Thioridazine<br>Thiothixene<br>Zuclopenthixol
Atypical Antipsychotics	Amisulpride<br>Aripiprazole<br>Clozapine<br>Olanzapine<br>Paliperidone<br>Quetiapine<br>Risperidone<br>Sulpiride<br>Ziprasidone<br>Zotepine
Prokinetics	Clebopride<br>Levosulpiride<br>Metoclopramide<br>Prochlorperazine<br>Sulpiride*
Calcium channel blockers	Cinnarizine<br>Diltiazem<br>Flunarizine<br>Verapamil
Anti-epileptics	Levetiracetam<br>Phenytoin<br>Valproate
Dopamine depeleters	Reserpine<br>Tetrabenazine

*Some products of sulpiride were approved as a prokinetic by Ministry of Food and Drug Safety, Republic of Korea.

International Journal of
Environmental Research and Public Health

MDPI

Table S3. *p*-Values for the interaction terms in the multivariate Cox proportional hazard regression model between trimetazidine use and covariates.

Variables*	*p*-Value
Sex	0.50
Age	0.88
Public Insurance Scheme	0.58
Residency	0.20
Diabetes	0.64
ESRD	0.40
Stroke	0.81
Calcium channel blockers	0.83
Prokinetics (or anti-emetics)	0.98
Typical antipsychotics	0.98
Atypical antipsychotics	0.28
Anti-epileptics	0.95

*These variables were the covariates with the multivariate-model *p*-value <0.05 in Table 2.

Table S4. Sensitivity analysis of adjusted hazard ratios of trimetazidine use for parkinsonism with shifting index date.

Index Date	No. of Subjects	Person-Years	No. of Events	Incidence Rate (Per 1000 Person-Years)	Adjusted HRs of Trimetazidine Use (95% CI)†	*p*-Value
*Shifting the index date, year, with respective eligible population and exposure ascertainment **						
January 1, 2007	4989	40,538	377	9.30	1.43 (1.26-1.61)	<0.0001
**January 1, 2008 (main)**	9712	70,840	662	9.35	1.38 (1.26-1.51)	<0.0001
January 1, 2009	14,253	92,061	844	9.17	1.35 (1.24-1.46)	<0.0001
January 1, 2010	18,787	105,032	969	9.23	1.37 (1.27-1.48)	<0.0001

*Eligible population was re-assessed according to the shifted index date, and the trimetazidine exposure, as well as other covariates were re-measured from the time-stamped database, so that the predictive variables were measured prior to the outcome ascertainment period. †Adjusted hazard ratios (95% confidence intervals) of trimetazidine use were calculated with a multivariate Cox proportional hazard model for parkinsonism with all covariates presented in Table 2. Abbreviation: CI, confidence interval; HR, hazard ratio; No, number.

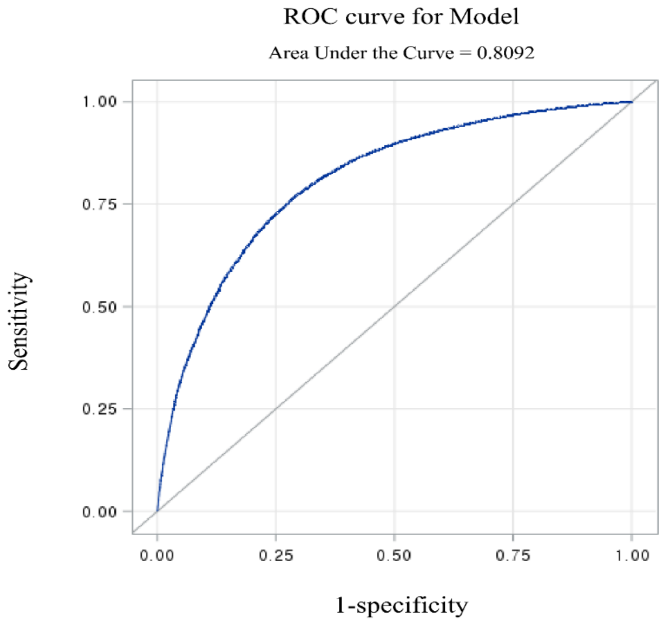

Figure S1. Receiver operating characteristics (ROC) curve for the propensity score model.

International Journal of
Environmental Research
and Public Health

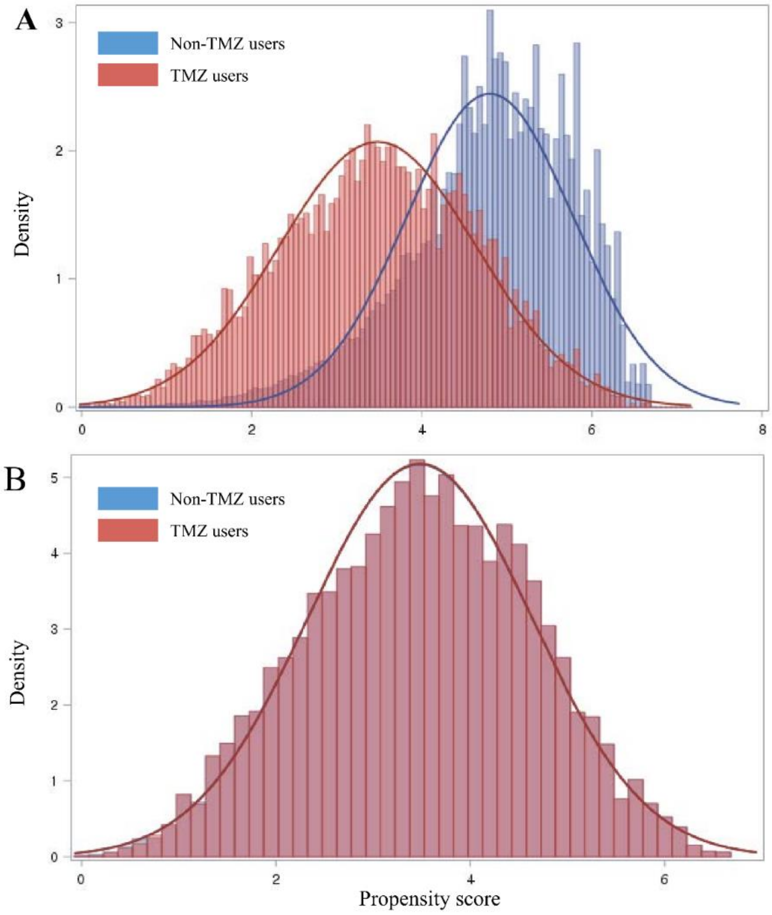

Figure S2. Distribution of propensity score between trimetazidine users and non-trimetazidine users before (2a) and after (2b) propensity score matching. TMZ, trimetazidine.

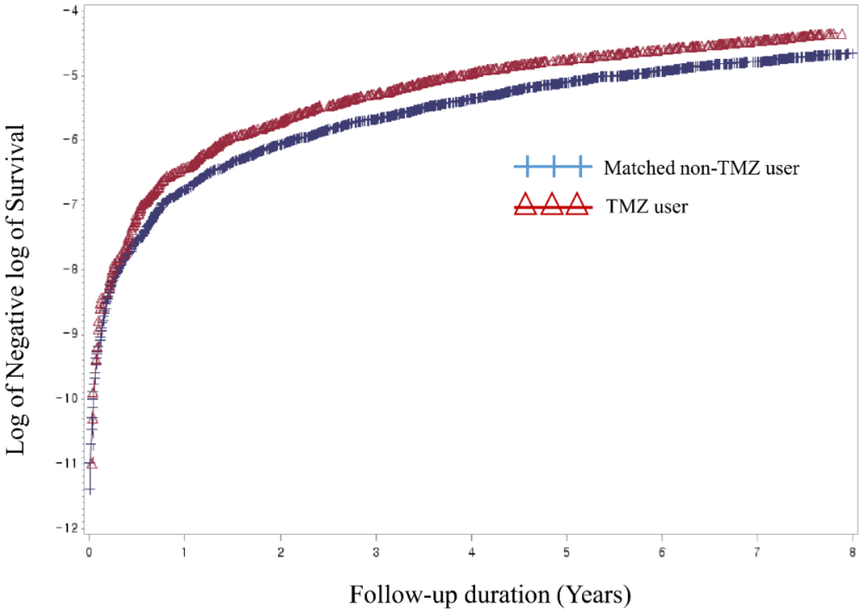

Figure S3. Log (minus log) curves for checking proportional hazard assumption. TMZ, trimetazidine.
